# Metachronous, colitis-associated rectal cancer that developed after sporadic adenocarcinoma in an adenoma in a patient with longstanding Crohn’s disease: a case report

**DOI:** 10.1186/1477-7819-11-295

**Published:** 2013-11-19

**Authors:** Hiroshi Takeyama, Tsunekazu Mizushima, Kiyokazu Nakajima, Mamoru Uemura, Naotsugu Haraguchi, Junichi Nishimura, Taishi Hata, Ichiro Takemasa, Hirofumi Yamamoto, Yuichiro Doki, Masaki Mori

**Affiliations:** 1 Department of Surgery, Gastroenterological Surgery, Graduate School of Medicine, Osaka University, 2-2 Yamada-Oka, Suita, Osaka 565-0871, Japan

**Keywords:** Colitis-associated cancer, Crohn’s disease, Sporadic cancer

## Abstract

**Background:**

Colorectal cancer associated with Crohn’s disease (CD) is increasing in proportion to the number of patients with CD in Japan. There are two subtypes of colorectal cancer with CD: sporadic cancer and colitis-associated cancer. Early diagnosis of colitis-associated cancer is sometimes difficult; when colorectal cancer is found in patients with CD, both colitis-associated cancer and sporadic cancer should be kept in mind. Here, we describe a case of metachronous, colitis-associated rectal cancer that developed after the complete resection of an adenoma that became a sporadic adenocarcinoma in a patient with longstanding CD. To the best of our knowledge, this is the first report of colitis-associated cancer in a patient with CD after removal of a sporadic cancer.

**Case presentation:**

We describe a 51-year old man with CD who had difficulty in defecation. A rectal polyp was detected and a transanal resection of the polyp was performed. A histopathological examination showed an adenoma with sporadic adenocarcinoma. After three years, a follow-up colonoscopy revealed a reddish, elevated lesion in the patient’s rectum. A colonoscopic biopsy showed a signet ring cell carcinoma. We performed an abdominoperineal resection of the rectum and a bilateral pelvic lymph node dissection. A histopathological examination revealed a mucinous adenocarcinoma with signet ring cell carcinoma and lymph node metastasis. The patient received adjuvant chemotherapy with oral uracil 224 mg combined with tegafur 100 mg plus leucovorin. No signs of recurrence were noted at a follow-up 18 months after the third surgery and 60 months after the second surgery.

## Background

Colorectal carcinoma (CRC) is a serious complication of inflammatory bowel disease (IBD). CRC accounts for approximately 15% of all deaths associated with IBD [1]. CRC has been thoroughly studied in conjunction with ulcerative colitis (UC) [2], but CRC is not well understood in conjunction with Crohn’s disease (CD). Several reports have described an increased incidence of CRC in CD [3-5]. However, relatively few studies have focused on CRCs in CD. No well-defined risk factors have been associated with the development of CRC in CD. Moreover, we lack clear recommendations for surveillance and surgical strategies for addressing CRC in CD [6]. CRC in patients with IBD can be classified into two subtypes, colitis-associated cancer and sporadic cancer, with different pathogenic and clinicopathological characteristics [6]. Few reports have precisely described each type of CRC in CD. To the best of our knowledge, no report has described metachronous sporadic and colitis-associated cancers in the same patient. This study is the first to report colitis-associated adenocarcinoma in a patient with CD after the removal of a sporadic adenocarcinoma.

## Case presentation

The patient was a 51-year old man with CD. He was initially diagnosed with CD at 23 years old. He had undergone ileocecal resection, owing to a stricture of the terminal ileum, at 36 years old, and the residual small intestine was 230 cm long. After the initial operation, he was prescribed 5-aminosalicylic acid and home parenteral nutrition. He did not accept a recommended colonoscopy examination, owing to anal pain.

**Figure 1:**
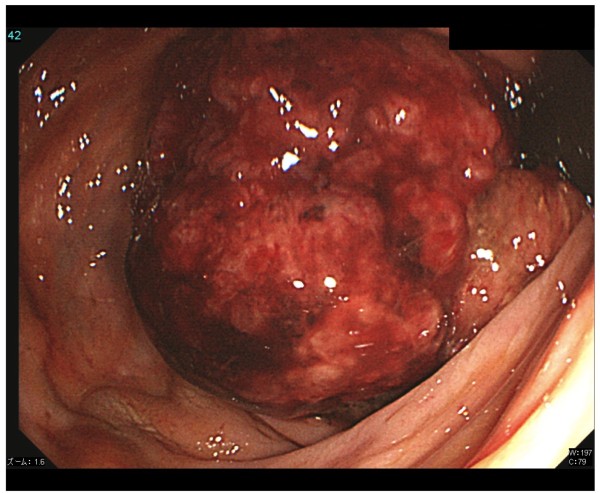
**Colonoscopic image shows a rectal polyp**, **4 cm in diameter.**

**Figure 2:**
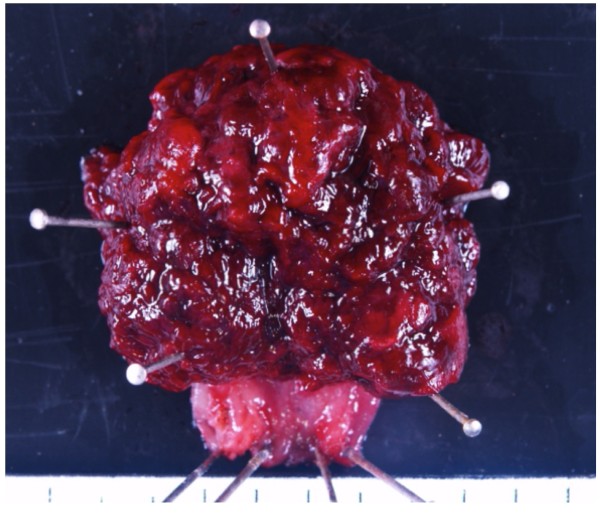
**Image of the surgical specimen shows the excised rectal polyp**, **4 cm in diameter.**

**Figure 3:**
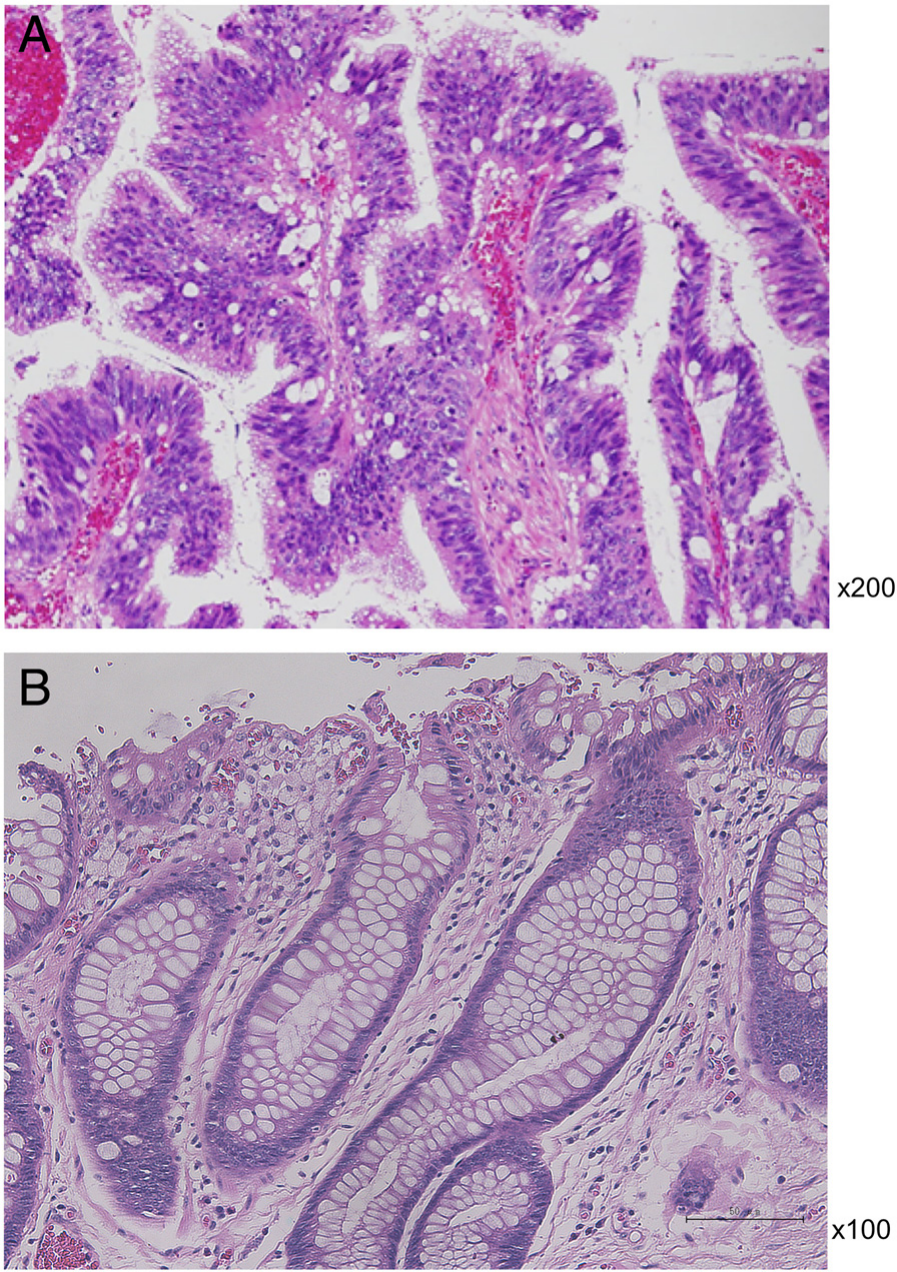
**Histopathological images. (A)** The polyp was diagnosed as sporadic adenocarcinoma in an adenoma, rather than colitis-associated adenocarcinoma (×200, H & E). **(B)** Section of tissue that bordered the polyp; there is no dysplasia or inflammation (×100, H & E stain).

**Figure 4:**
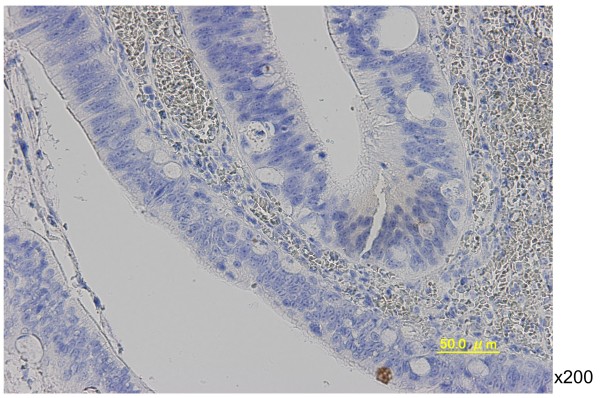
**Immunohistochemical staining for p53 in rectal mucosa tissue shows no dysplasia in the crypt base.** These findings were not consistent with a colitis-associated colorectal cancer (×200).

In August 2008, the patient complained of difficulty in defecation and consulted a physician at our hospital. A colonoscopy examination showed a pedunculated rectal polyp, 4 cm in diameter (Figure 1) and an anal stricture due to proctitis. Histological examination of the biopsy from the lesion showed an adenoma; no dysplasia was detected around the polyp. A transanal resection of the polyp was performed for treatment and final diagnosis (Figure 2). The pathological findings revealed that the polyp was an adenoma with well-differentiated adenocarcinoma without lymphatic or vascular invasion with negative margin of cut end, and that no dysplasia or inflammation was present around the polyp; thus, the polyp was diagnosed as a sporadic adenocarcinoma in an adenoma, rather than a colitis-associated adenocarcinoma (Figure 3). Consistent with the results of the biopsy, an endoscopic exploration showed no dysplasia in the rectal mucosa, and immunohistochemistry showed no p53-staining of the crypt base. These findings were not consistent with colitis-associated CRC (Figure 4). In July 2010, follow-up colonoscopy showed a longitudinal ulcer scar from the descending colon to the ascending colon and stenosis of the ileocolic anastomosis. We did not find any apparent lesion in the rectum.

**Figure 5:**
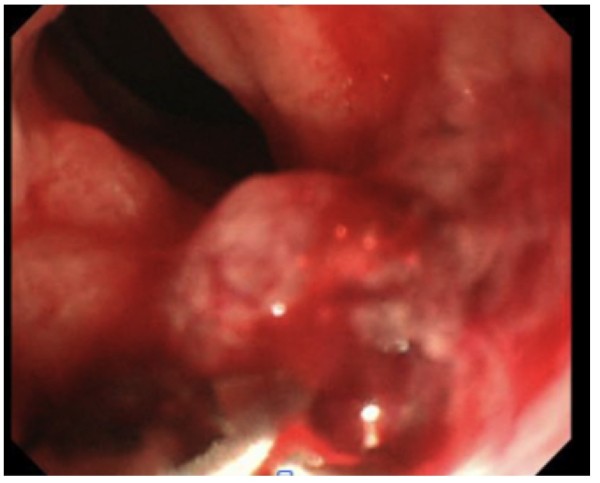
**Colonoscopic image shows a reddish**, **elevated lesion in the rectum.** This was located in a site different site from the initial lesion.

**Figure 6:**
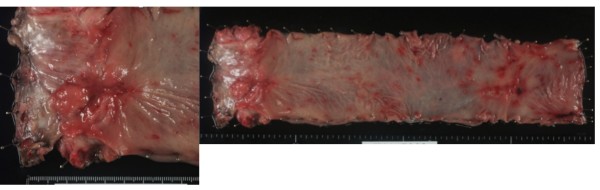
Image of the surgical specimen shows a reddish and elevated lesion, 3cm in diameter.

**Figure 7:**
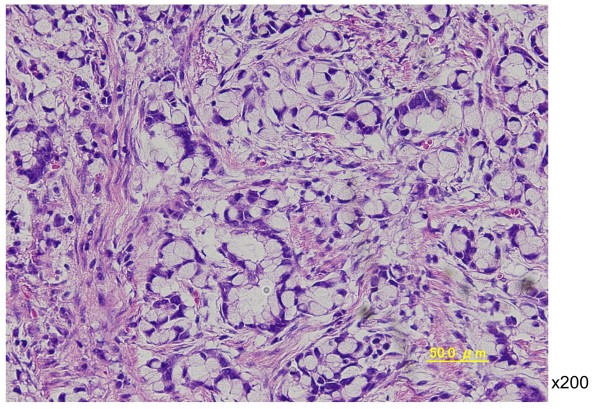
**Histological image of a postoperative specimen shows a mucinous adenocarcinoma with signet ring cell carcinoma.** (×200, H & E stain).

**Figure 8:**
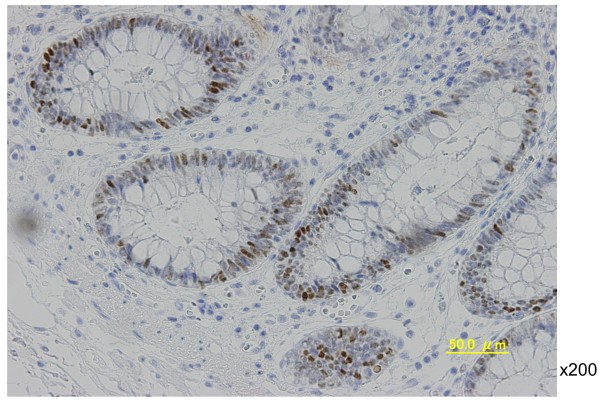
**Immunohistochemical staining for p53 in rectal mucosa tissue shows dysplasia in the crypt base**, **consistent with colitis**-**associated colorectal cancer.** (×200).

Three years after the second surgery, in August 2011, colonoscopy examination revealed a reddish, elevated lesion in the rectum (Figure 5). This lesion was located at a different site from the initial lesion. A colonoscopic biopsy revealed a signet ring cell carcinoma. We performed an abdominoperineal resection of the rectum and a bilateral pelvic lymph node dissection (Figure 6). Postoperative histological examination showed a mucinous adenocarcinoma with signet ring cell carcinoma and lymph node metastasis (Figure 7). Dysplasia was detected in the rectal mucosa, and the crypt base was immunohistochemically stained with p53. These findings were consistent with colitis-associated CRC (Figure 8).

Postoperatively, the patient received adjuvant chemotherapy with 224 mg oral uracil combined with100 mg tegafur plus leucovorin for 6 months. The patient has continued with regular follow-ups over the last 18 months after the third surgery and over the last 60 months after the second surgery. He has also undergone regular examinations. A regular colonoscopy examination was performed every 6 months, and no relapse of colonic inflammation or dysplasia was detected.

## Discussion

CRC accounts for approximately 15% of all deaths in patients with IBD [6,7]. The risk of CRC for patients with IBD increases by 0.5% to 1% yearly from 8 to 10 years after the IBD diagnosis. A meta-analysis of published studies that focused on the CRC risk in UC showed that the risk of CRC for patients with colitis was 2% at 10 years, 8% at 20 years, and 18% after 30 years of colitis [8]. Previously, CD was not considered a risk factor for CRC; however, recent studies have shown that the CRC risk in patients with colonic CD is similar to that of patients with UC [7,9].

In patients with IBD, CRC can take the form of sporadic cancer or colitis-associated cancer. In patients with UC who develop colitis-associated cancer, total proctocolectomy is the standard treatment; however, when sporadic cancer occurs, total proctocolectomy is not always necessary. In patients with CD, an indication for total proctocolectomy has been controversial. However, it was also reported that, when a diagnosis of colitis-associated cancer is known in advance of surgery, a total proctocolectomy should be seriously considered, to eliminate the risk of metachronous disease. In 44% of these patients, dysplasia occurred at a site remote from the site of the cancer [10]. However, this patient had already undergone ileocecal resection and the residual small intestine was 230 cm long, resulting in intestinal failure syndrome requiring home parental nutrition. For these reasons, we chose abdominoperineal resection with colostomy, not total proctocolectomy with ileostomy, in order to preserve the colon and maintain absorption of water and electrolytes. After the last surgery, we conducted intensive cancer surveillance and neither a recurrence nor another lesion has been detected at this time.

However, the site of inflammation should be resected, owing to the risk of dysplasia. When the colon and rectum are preserved in surgery, a scheduled, annual surveillance with biopsies is strongly recommended [10]. It is very important, when planning therapy and surveillance after surgery, to perform a differential diagnosis to distinguish between colitis-associated CRC and sporadic CRC. However, it is often difficult to achieve this differential diagnosis in patients with IBD, particularly before surgery [11,12].

The American Gastroenterological Association and the British Society for Gastroenterology surveillance guidelines have recommended beginning surveillance after 8 to 10 years of disease in cases of CD or extensive UC, and after 15 to 20 years of disease in cases of left-sided UC [13-15]. However, the evidence on which this is based on is poor. It was reported that cancer has occurred in a substantial number of patients with IBD (17% to 28%) before the recommended time intervals for surveillance [16].

Clinically, endoscopic examination is the most useful screening method. However, even with endoscopic examination, it is often difficult to detect dysplastic changes. Colitis-associated dysplasia may show subtle macroscopic features that mimic a broad variety of lesions, ranging from inflammation to carcinoma. Previous reports have indicated that dysplastic lesions were not visible upon endoscopy in 50% to 80% of cases with colitis-associated cancers [17]. Conversely, sporadic cancer mainly develops from polyps that are on the pathway to becoming adenoma-carcinomas, and a polyp is easily detectable, even in a normal endoscopy examination. In contrast, the gross appearance of colitis-associated neoplasms varies from case to case, as the neoplasm develops from dysplasia to become a carcinoma. Dysplastic lesions may be irregularly delineated, plaque-like, irregularly elevated, or verrucous structures; therefore, they are difficult to identify with normal endoscopy [18].

This case should make surgeons and endoscopists aware of the possibility that patients with CD may develop either colitis-associated cancer or sporadic cancer. This report should alert surgeons and endoscopists to look for colitis-associated cancers that are in a precancerous or early stage. In this case study, although we performed annual colonoscopic surveillance, we did not detect the second lesion, the colitis-associated cancer, until it reached an advanced cancerous stage. Early detection is highly important for avoiding surgeries, including colectomy or proctectomy. However, it is often difficult to detect colitis-associated cancers in dysplastic, precancerous, or early stages. We need further investigation to discover novel biomarkers that make it possible to detect cancer in blood samples or biopsies.

## Conclusions

To the best of our knowledge, this is the first case report of a metachronous colitis-associated rectal cancer that arose in a patient with CD after removal of a sporadic cancer. This case emphasized the importance for clinicians to bear in mind the possibilities of both sporadic cancer and colitis-associated cancer when following-up patients with CD. It is also important to consider surgical therapy and surveillance after surgery, and to perform a differential diagnosis to distinguish between colitis-associated cancer and sporadic cancer. CRC associated with CD remains a rare occurrence, and there is no defined surveillance strategy. However, patients with longstanding CD should receive cancer surveillance as frequently as do patients with UC.

## Consent

Written informed consent was obtained from the patient for publication of this case report and accompanying images. A copy of the written consent is available for review by the editor-in-chief of this journal.

## Abbreviations

CD: Crohn’s disease; CRC: Colorectal carcinoma; H & E: Hematoxylin and eosin; IBD: Inflammatory bowel disease; UC: Ulcerative colitis.

## Competing interests

There are no conflicts of interest to disclose for any author.

## Authors’ contributions

HT wrote the main manuscript, and TM and KN performed the operation, revised the manuscript for important intellectual content, and gave the final approval for the version to be submitted for publication. All authors read and approve the final manuscript.
